# The Brussels Health Congress

**Published:** 1920-05-01

**Authors:** Gladys M. E. Leigh


*To the Editor of* THE HOSPITAL.

### The Brussels Health Congress.

*To the Editor of* THE HOSPITAL.

SIR,—Through the medium of the nursing press the Royal Institute of Public Health announces the programme of the Brussels Conference. In the State Medicine Section there will be English speakers on infant welfare, eye diseases, &c. Dame Mary Scharlieb, G.B.E., M.D., M.S., will preside over Section V., Hygiene and Women's Work, to which various women will contribute papers, but there is no nurse speaker. For years and years and years the nursing services have been the pioneers in the field of women's work. During long hours they have worked through shine and shower, in heat and cold, often under appalling conditions, badly housed, and shamefully remunerated, they have been the stepping-stones on which health visitors and infant welfare workers have risen to more entrancing heights. Are they no longer of any service in the land? Does the Royal Institute of Public Health consider it can afford to exclude the nursing services from an important conference as a negligible factor in the national health, or is the omission merely due to thoughtlessness? Amongst the heads of the nursing services there are several excellent speakers, have they nothing to contribute to the conference which might be either interesting or instructive? Have they been asked to do so, and have they declined, or have they been merely ignored?

Yours faithfully,
GLADYS M. E. LEIGH.

